# Double-Edged Sword: HBV-Driven Lipid Metabolism Remodeling in Hepatocarcinogenesis Through Ferroptosis Regulation

**DOI:** 10.3390/v18101075

**Published:** 2026-09-29

**Authors:** Sihan Xia, Xueqin Shen, Guangyu Chen, Ruifang Liu, Xin Yang

**Affiliations:** 1 The Institutes of Biology and Medical Sciences, Suzhou Medical College, Soochow University, Suzhou 215123, China; 20244252024@stu.suda.edu.cn (S.X.); 20244252002@stu.suda.edu.cn (X.S.); 20245252008@stu.suda.edu.cn (G.C.); 2 Biomedical Basic Research Center (BBRC) of Jiangsu, Soochow University, Suzhou 215123, China; 3 Jiangsu Key Laboratory of Infection and Immunity, Soochow University, Suzhou 215123, China; 4 Minister of Education Key Laboratory of Geriatric Diseases and Immunology, Soochow University, Suzhou 215123, China

**Keywords:** HBV, HBx, hepatocarcinogenesis, HCC, lipid metabolism, ferroptosis, immune microenvironment, inflammation

## Abstract

Chronic hepatitis B virus (HBV) infection remains a leading cause of hepatocellular carcinoma (HCC) worldwide, particularly in Asia. Although nucleos(t)ide analogs suppress viral replication and reduce HCC incidence, residual risk persists, highlighting the need to better understand HBV-driven hepatocarcinogenesis. HBV alters host transcription, DNA repair, lipid metabolism, and the hepatic immune microenvironment. These changes help sustain liver injury, fibrogenesis, and malignant transformation. Ferroptosis, an iron-dependent form of programmed cell death driven by excessive lipid peroxidation, has emerged as an important mechanism in this context, because the liver is central to iron and lipid metabolism. Here, we propose that HBV-driven lipid remodeling shapes ferroptosis susceptibility in a stage-dependent, double-edged manner. During chronic infection, oxidative stress and enrichment of peroxidation-prone lipid substrates may promote ferroptosis-associated hepatocyte injury, immune dysregulation, and fibrosis. After malignant transformation, HBV-related oncogenic programs may instead favor ferroptosis evasion through altered lipid composition and strengthened antioxidant defenses, thereby supporting tumor survival and progression. In this review, we summarize mechanisms linking HBV, lipid metabolic remodeling, and ferroptosis while distinguishing direct HBV-related evidence from findings inferred from other liver disease or cancer models. This HBV-ferroptosis framework may help refine risk stratification and suggest new therapeutic opportunities in HBV-related HCC.

## 1. Introduction

Persistent hepatitis B virus (HBV) infection remains a major global health challenge and a leading cause of hepatocellular carcinoma (HCC), especially in Asia [1,2]. Despite the availability of effective vaccination and antiviral therapy, HBV continues to account for substantial cirrhosis- and liver cancer-related mortality [1,2]. Chronic HBV infection follows a dynamic disease continuum that may progress from hepatitis to fibrosis, cirrhosis, and ultimately HCC [1,2,3]. Large prospective studies have shown that HCC risk is closely associated with viral activity. Serum HBV DNA levels correlate with HCC incidence in a biological gradient, and high hepatitis B surface antigen (HBsAg) levels remain predictive of HCC even in patients with relatively low viral loads [3,4]. Importantly, although nucleos(t)ide analog therapy substantially lowers HCC risk, baseline viral characteristics continue to influence on-treatment liver cancer risk, indicating that long-term virological suppression does not fully eliminate the risk of HBV-related hepatocarcinogenesis [5]. This underscores the complexity of HBV-driven liver cancer and suggests that mechanisms beyond viral replication also contribute to HCC progression.

Accumulating evidence indicates that HBV-host crosstalk can trigger inflammatory cell death, including apoptosis, necroptosis, pyroptosis, and potentially ferroptosis. These processes may contribute to chronic liver injury and creating a microenvironment permissive for fibrogenesis and hepatocarcinogenesis [6]. Indeed, dying hepatocytes release damage-associated molecular patterns (DAMPs), inflammatory cytokines, and lipid peroxidation products, which activate resident and infiltrating myeloid cells, including Kupffer cells and neutrophils, as well as hepatic stellate cells (HSCs) [6,7]. Mederacke et al. demonstrated that P2Y purinoceptor 14 (P2Y14) is a molecular link connecting hepatocyte death to HSC activation and fibrogenesis [8]. Furthermore, mitochondria-derived danger signals, primarily mitochondrial DNA (mtDNA), can be released from injured hepatocytes and promote HSC activation and liver fibrosis [9]. Lipid peroxidation-derived aldehydes, such as 4-hydroxynonenal (4-HNE), can also function as a profibrogenic stimulus in activated human HSCs [10]. Notably, during chronic HBV infection, ferroptosis-related dysregulation is not restricted to hepatocytes. Targeting ferroptosis can restore the antiviral activity of CD8^+^ T cells, further linking ferroptosis to viral persistence and immune dysfunction [11]. Collectively, these findings suggest that inflammatory cell death in hepatocytes may not be merely a downstream consequence of chronic HBV infection but contribute to liver microenvironment remodeling, fibrosis, immune dysregulation, and ultimately HCC initiation (Table 1).

**Figure 1:**
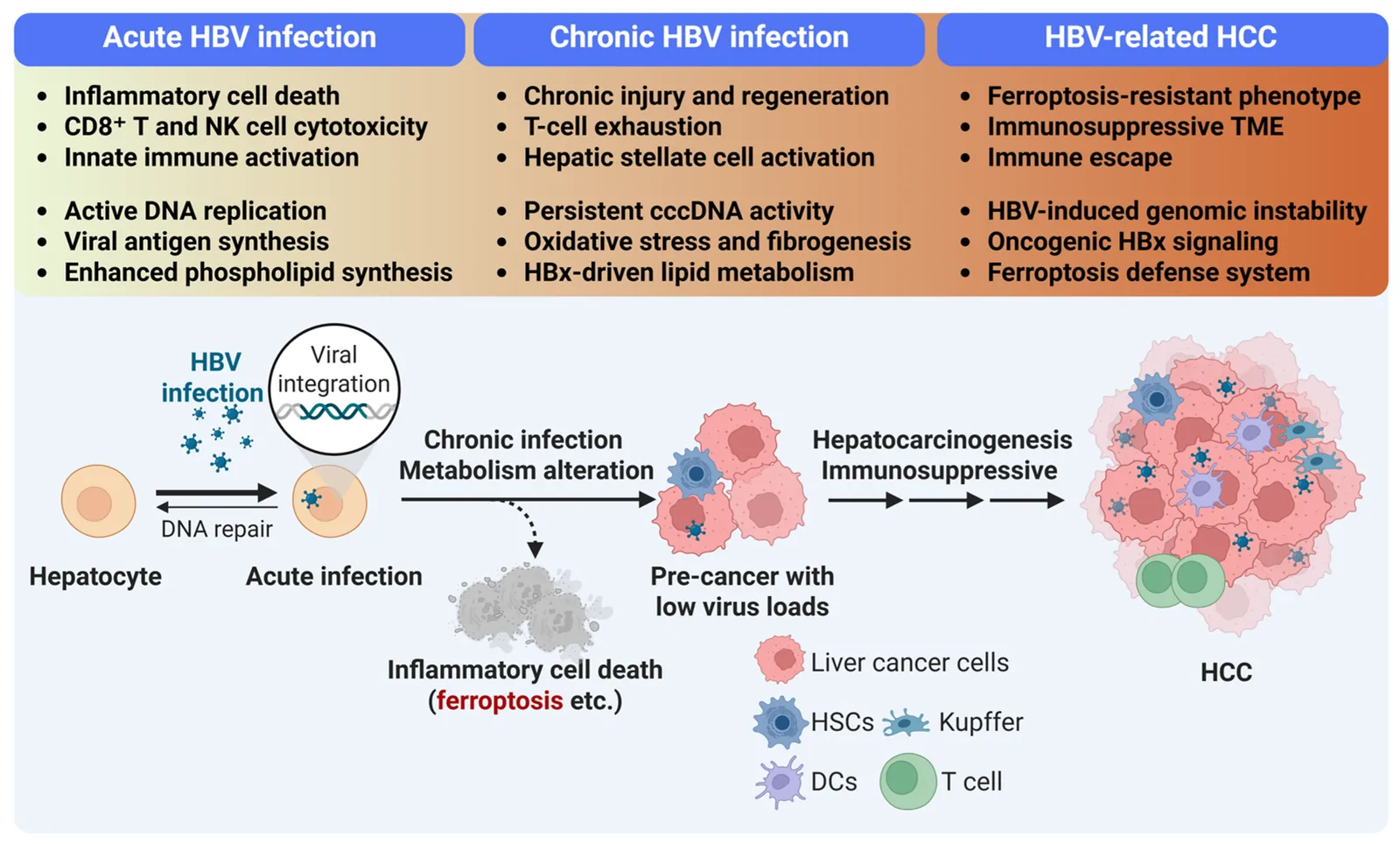
Dynamic progression from HBV infection to HBV-related HCC. This schematic illustrates the stage-dependent pathological and molecular events during HBV-related HCC. In the acute infection stage, HBV infects hepatocytes and undergoes active DNA replication and viral antigen synthesis, accompanied by enhanced phospholipid synthesis to support viral replication. Infected hepatocytes are eliminated through innate immune activation, CD8^+^ T-cell and NK-cell cytotoxicity, and inflammatory cell death, including ferroptosis. During chronic HBV infection, incomplete viral clearance results in persistent cccDNA activity, chronic liver injury and compensatory regeneration. HBx-driven lipid metabolic remodeling and hepatic stellate cell activation-mediated fibrogenesis contribute to a precancerous microenvironment. In the HCC stage, HBV DNA integration, oncogenic HBx signaling, genomic instability and activation of ferroptosis defense systems promote a ferroptosis-resistant tumor phenotype. Abbreviations: HBV, hepatitis B virus; HCC, hepatocellular carcinoma; cccDNA, covalently closed circular DNA; HBx, hepatitis B virus X protein; TME, tumor microenvironment; HSCs, hepatic stellate cells; DCs, dendritic cells. Created in BioRender. Yang, X. (2026) https://BioRender.com/9b9go9k.

Previous discussions have often considered HBV-driven metabolic alterations and ferroptosis separately. In contrast, this review proposes that HBV-related lipid remodeling may provide a mechanistic bridge linking viral persistence, chronic inflammatory liver injury, malignant transformation, and tumor cell survival. In this framework, ferroptosis is not uniformly detrimental or protective. Rather, its biological consequence depends on disease stage, lipid substrate availability, antioxidant capacity, and the responding cell type. HBV-driven lipid remodeling may therefore act as a “double-edged sword”: it may promote ferroptosis-related hepatocyte injury and inflammatory remodeling during chronic infection, while later favoring ferroptosis evasion and malignant cell persistence in established HBV-related HCC. Because direct mechanistic evidence connecting specific HBV-driven lipid changes to ferroptosis remains incomplete, throughout this review, we distinguish evidence derived directly from HBV/HBx-related experimental systems from indirect evidence in HBV-related disease contexts and extrapolated findings from non-HBV HCC or other disease models (Figure 1).

## 2. Brief Summary of HBV Biology and Pathogenesis

### 2.1. Molecular Architecture and Replication Cycle

HBV is a small enveloped DNA virus that preferentially infects hepatocytes [32]. In addition to receptor-dependent entry, HBV surface proteins interact with heparan sulfate proteoglycans (HSPGs, including syndecans and glypicans) on the hepatocyte surface, a process that is critical for infectivity [33]. Moreover, sodium taurocholate cotransporting polypeptide (NTCP) is the functional receptor required for HBV entry [34]. The HBV genome is present in nucleocapsids primarily as relaxed circular DNA (rcDNA) [35], which is delivered to the nucleus after viral entry and repaired into covalently closed circular DNA (cccDNA) [36].

This cccDNA serves as the transcriptional template for all viral RNAs, including pregenomic RNA (pgRNA) and subgenomic RNAs encoding HBV surface proteins, core proteins, polymerases, and hepatitis B virus X protein (HBx) [37]. cccDNA-mediated transcription is highly dependent on both viral and host factors. HBx plays a central role in maintaining active cccDNA transcription by counteracting the host restriction complex, structural maintenance of chromosomes protein 5/6 (SMC5/6) [38,39]. Interestingly, metabolic enzymes and metabolites have also been implicated in cccDNA regulation. For example, glutamate dehydrogenase 1-dependent α-ketoglutarate promotes HBV transcription by modulating histone methylation on the cccDNA minichromosome [40]. Following transcription, pregenomic RNA (pgRNA) is reverse transcribed into rcDNA within newly formed nucleocapsids. Mature nucleocapsids either acquire an envelope and are secreted as progeny virions or recycle their rcDNA back to the nucleus to replenish the cccDNA pool [32,41].

### 2.2. HBV-Related Hepatocarcinogenesis

HBV promotes hepatocarcinogenesis through several non-mutually exclusive mechanisms, especially HBx-driven oncogenic signaling, chronic inflammation, and metabolic reprogramming [7,42,43]. Large-scale genomic analyses of HCC have revealed recurrent HBV integration events and broad genomic alterations in HBV-related tumors [44]. HBV DNA integration can disrupt host genes, promote chromosomal instability, generate viral-host fusion transcripts, and sustain the expression of viral proteins such as HBx or truncated HBsAg even when productive replication is limited [45]. HBx is one of the most intensively studied viral oncoproteins. As mentioned above, HBx promotes cccDNA transcription by antagonizing SMC5/6, and therefore impairs homologous recombination and genomic stability [38,46,47]. HBx also activates oncogenic pathways such as mechanistic target of rapamycin (mTOR) and vascular endothelial growth factor A (VEGFA) signaling, thereby promoting angiogenesis and inflammatory transformation [48]. C-terminally truncated HBx, frequently generated through HBV integration, has been associated with invasion, metastasis, and metabolic reprogramming in HCC [49,50].

Chronic HBV infection also contributes to a fibrogenic and pro-tumorigenic hepatic milieu. HBV can induce liver fibrosis through transforming growth factor beta 1 (TGF-β1)-related pathways and through activation of HSCs [51,52]. HBV-related oxidative stress, and nuclear factor kappa-light-chain-enhancer of activated B cells (NF-κB) activation further contribute to fibrogenesis and malignant progression [10,16,53,54]. Therefore, HBV-related liver cancer should be viewed as the consequence of both cell-intrinsic viral effects and long-term remodeling of the liver microenvironment.

### 2.3. Metabolic Reprogramming upon HBV Infection

Metabolic reprogramming is a central feature of HBV infection and HBV-related liver disease. HBV replication is sensitive to cellular nutrient status and energy-sensing pathways. Glucose availability regulates HBV replication and autophagy through AMP-activated protein kinase (AMPK) and mTOR signaling [55]. More broadly, HBV has been described as a “metabolic virus” because it reshapes host glucose and lipid metabolism in ways that may support viral persistence and disease progression [56,57]. Moreover, HBV can directly also exploit host transcriptional regulators, such as peroxisome proliferator-activated receptor alpha (PPARα), to favor viral transcription [58].

Metabolomic studies further show that cirrhosis and HBV-related HCC are associated with distinct metabolic signatures [59,60]. Lipid metabolism-related gene signatures have prognostic value in HBV-related HCC [61]. Importantly, HBV-related metabolic reprogramming may also reshape the cellular metabolome to evade innate immune recognition [62]. Yu et al. showed that chronic HBV infection impairs natural killer (NK) cell glucose metabolism and effector function through the HBsAg/interleukin-15/mTOR axis [63]. Other studies suggest that metabolic reprogramming contributes to CD8^+^ T-cell exhaustion during chronic infection [64]. Thus, HBV-driven metabolic reprogramming may connect viral replication, immune dysfunction and malignant transformation.

## 3. Lipid Metabolism and Ferroptosis

**Figure 2:**
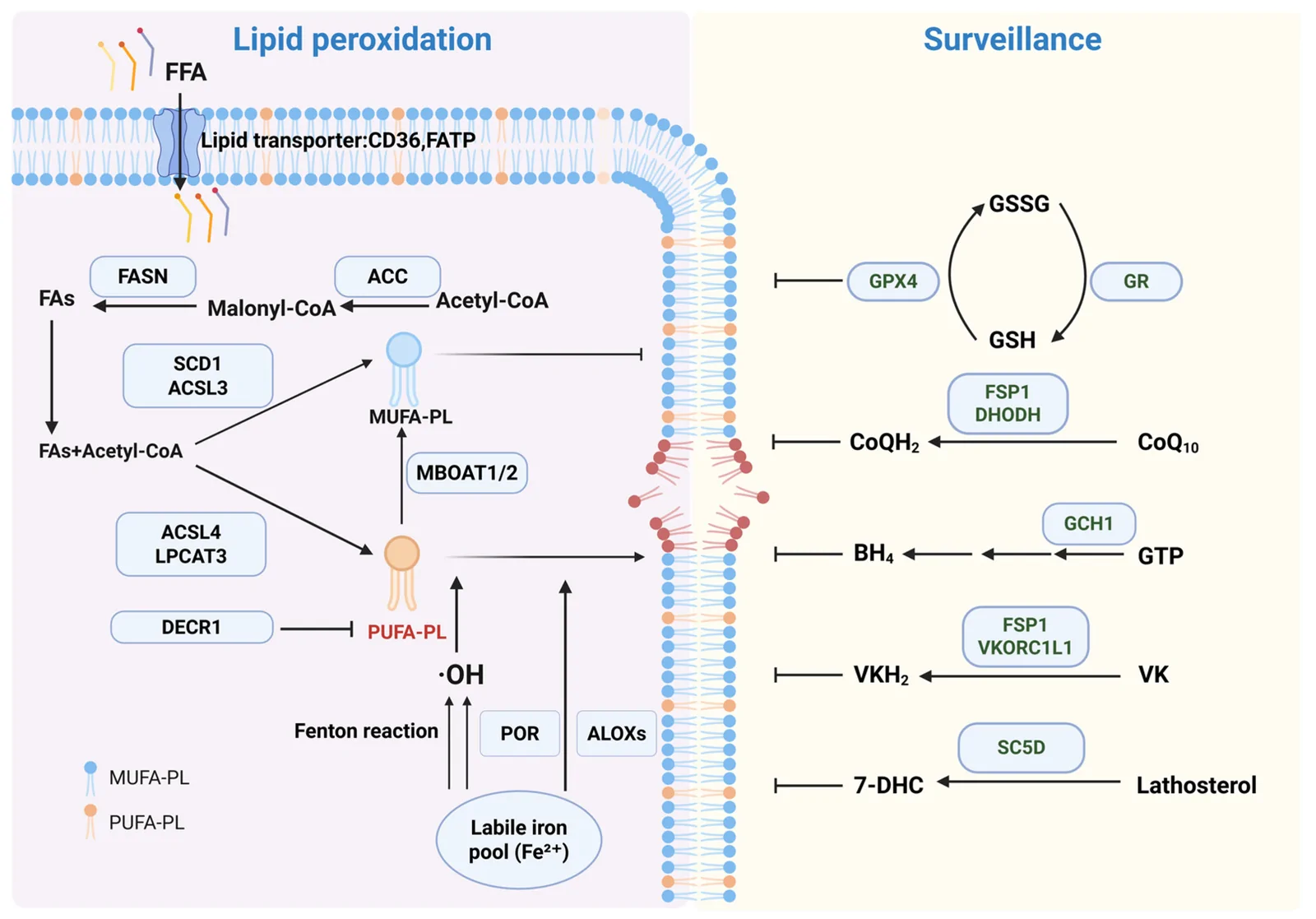
Lipid metabolic determinants of ferroptosis and ferroptosis surveillance systems. This schematic summarizes how lipid metabolism controls ferroptosis susceptibility through the balance between lipid peroxide generation and antioxidant defense. SCD1 and ACSL3 promote the formation of MUFA-PL, which generally reduce ferroptosis sensitivity. In contrast, ACSL4 and LPCAT3 facilitate the incorporation of polyunsaturated fatty acids into phospholipids. Lipid peroxi-dation is amplified by enzymatic reactions or non-enzymatic Fenton reactions driven by the labile iron pool. On the right panel, multiple ferroptosis surveillance systems suppress excessive lipid peroxidation. In parallel with canonical GSH/GPX4 axis, FSP1 and DHODH maintain the reduced CoQH2 pool, which functions as a lipid-soluble antioxidant. The GCH1–BH4 pathway and VKORC1L1- and FSP1-mediated vitamin K reduction pathway provide additional radical-trapping antioxidant systems. Abbreviations: FFA, free fatty acid; FABP, fatty acid-binding protein; ACC1, acetyl-CoA carboxylase 1; FASN, fatty acid synthase; SCD1, stearoyl-CoA desaturase 1; ACSL3/4, acyl-CoA synthetase long-chain family member 3/4; LPCAT3, lysophosphatidylcholine acyltrans-ferase 3; MBOAT1/2, membrane-bound O-acyltransferase domain-containing 1/2; DECR1, 2,4-dienoyl-CoA reductase 1; MUFA-PL, monounsaturated fatty acid-containing phospholipid; PUFA-PL, polyunsaturated fatty acid-containing phospholipid; ALOXs, arachidonate lipoxygenas-es; POR, cytochrome P450 oxidoreductase; GPX4, glutathione peroxidase 4; GR, glutathione re-ductase; FSP1, ferroptosis suppressor protein 1; DHODH, dihydroorotate dehydrogenase; GCH1, GTP cyclohydrolase 1; VKORC1L1, vitamin K epoxide reductase complex subunit 1-like 1; SC5D, sterol-C5-desaturase. Created in BioRender. Yang, X. (2026) https://BioRender.com/ygz3xg5.

Ferroptosis is an iron-dependent form of regulated non-apoptotic cell death characterized by the lethal accumulation of lipid peroxides on cellular membranes [65]. Unlike apoptosis, necroptosis, or pyroptosis, ferroptosis is primarily governed by the balance between iron-catalyzed lipid oxidation and lipid peroxide detoxification systems [66]. Since its discovery, ferroptosis has been linked to a wide range of human diseases, including degenerative disorders, infectious diseases, and cancer [67]. Accumulating evidence suggests that ferroptosis is an important mechanism in liver diseases [68]. Indeed, at the molecular level, ferroptosis can be understood as a process in which susceptible lipid substrates, redox-active iron, and impaired antioxidant defenses converge to trigger catastrophic membrane damage (Figure 2).

### 3.1. Lipid Peroxidation

Lipid peroxidation is the central execution mechanism of ferroptosis, which depends on three interrelated factors: the abundance of lipids containing polyunsaturated fatty acids (PUFAs), iron-dependent enzymatic or non-enzymatic oxidation and the capacity of cells to detoxify lipid peroxides.

The most ferroptosis-prone lipid species are polyunsaturated fatty acid-containing phospholipids (PUFA-PLs), because bis-allylic hydrogen atoms in PUFAs are highly susceptible to radical-mediated oxidation [69,70,71]. Oxidized arachidonic acid- and adrenic acid-containing phosphatidylethanolamines have been identified as important death signals that drive ferroptotic cell death. Acyl-CoA synthetase long-chain family member 4 (ACSL4) cooperates with lysophosphatidylcholine acyltransferase 3 (LPCAT3) to promote the incorporation of PUFAs into phospholipids, thereby shaping the cellular lipid pool toward a ferroptosis-sensitive state [70,71]. In addition, phospholipids bearing two polyunsaturated fatty acyl tails can strongly promote ferroptosis, emphasizing that both fatty acyl composition and phospholipid headgroup context determine ferroptosis sensitivity [72,73]. Conversely, exogenous monounsaturated fatty acids (MUFAs) can induce an ACSL3-mediated ferroptosis-resistant state by competing with PUFAs for incorporation into phospholipids [74]. Indeed, the PUFA-to-MUFA ratio is an important determinant of cellular susceptibility to ferroptosis. For example, lysophosphatidylcholine acyltransferase 1 (LPCAT1) increases membrane phospholipid saturation via the Lands cycle to protect cells from lipid peroxidation and inhibit ferroptosis [75]. Sex hormone regulated membrane-bound glycerophospholipid O-acyltransferase 1/2 (MBOAT1/2) also inhibit ferroptosis by remodeling the cellular phospholipid profile [76]. Similarly, 2,4-dienoyl-CoA reductase 1 (DECR1) reprograms the PUFA-to-MUFA ratio to repress ferroptosis induction [77,78]. Beyond the PUFA-to-MUFA ratio, membrane lipid architecture further determines whether lipid peroxidation culminates in ferroptotic death. Ether lipids can alter the distribution and oxidation potential of cellular lipids [79,80]. Furthermore, lipid droplets may function as buffering organelles that sequester oxidizable or peroxidized lipids, thereby limiting membrane damage [81] (Figure 2, left panel).

As the fundamental driver of ferroptosis, iron promotes lipid peroxidation through non-enzymatic or enzymatic oxidation pathways. Non-enzymatic oxidation, namely the Fenton reaction, generates highly reactive alkoxyl and/or hydroxyl radicals by the decomposition of hydrogen peroxide using ferrous iron (Fe^2+^). This process consists of three stages—initiation, propagation, and termination—and leads to the formation of hydroperoxide phospholipids (PLOOHs). The hydroxyl radicals attack the carbon-carbon double bonds (C=C) of PUFA-PLs to generate a phospholipid radical (PL•). Then, PL• reacts with oxygen to generate a phospholipid peroxyl radical (PLOO•) in the propagation step: PLOO• reacts with the adjacent PUFA-PL to produce PLOOH and another PL•. Thus, unless PLOOH is efficiently eliminated, lipid peroxides accumulate and eventually trigger ferroptosis. In addition to non-enzymatic oxidation, enzymatic lipid peroxidation also contributes to ferroptosis. Lipoxygenases can catalyze PUFA peroxidation and drive ferroptotic death [69], while arachidonate lipoxygenase ALOX12 has been linked to p53-mediated tumor suppression through ferroptosis induction [82]. Recent studies indicate that pleckstrin homology-like domain family A member 2 (PHLDA2) acts as a critical ferroptosis initiator by interacting with ALOX12 and promoting ALOX12-mediated phosphatidic acid peroxidation [83,84]. In addition, cytochrome P450 oxidoreductase (POR) also contributes to phospholipid peroxidation during ferroptosis [85].

The dependence of ferroptosis on PUFA-PL oxidation further underscores the importance of iron homeostasis. It is well-established that transferrin-mediated iron uptake and glutaminolysis can promote ferroptosis [86], whereas iron storage and iron mobilization critically influence the availability of redox-active iron. Autophagy can enhance ferroptosis by degrading ferritin, a process often referred to as ferritinophagy, thereby increasing the labile iron pool and promoting lipid peroxidation [87,88,89]. Iron-sulfur cluster deficiency, iron regulatory protein 2 (IRP2) signaling, glycogen synthase kinase 3 beta (GSK-3β) signaling, the nuclear factor erythroid 2-related factor 2 (NRF2)/E3 ubiquitin-protein ligase HERC2/vesicle-associated membrane protein 8 (VAMP8) axis, and the AKT/transient receptor potential mucolipin 1 (TRPML1)/ADP-ribosylation factor-like protein 8B (ARL8b) axis have all been implicated in the regulation of iron homeostasis and ferroptosis [90,91,92,93].

### 3.2. Anti-Ferroptosis Systems

On the other hand, cells have evolved multiple anti-ferroptosis systems to prevent lethal lipid peroxide accumulation. Solute carrier family 7 member 11 (SLC7A11, also known as xCT) imports cystine to enable the synthesis of a critical antioxidant, glutathione (GSH) [94]. Glutathione peroxidase 4 (GPX4) then uses GSH to reduce phospholipid hydroperoxides to their corresponding alcohols [95]. Pharmacological inhibition of SLC7A11 induces endoplasmic reticulum stress and ferroptosis [96], while genetic or functional inactivation of GPX4 triggers ferroptotic tissue injury in vivo [97]. Liu et al. identified HBx as a critical factor in potentiating D-galactosamine (D-GalN)-induced liver injury and ferroptosis by suppressing SLC7A11 expression, providing direct evidence that HBx promotes liver failure at least in part through ferroptotic hepatocyte death [12]. Thus, the SLC7A11/GSH/GPX4 axis is widely considered a central suppressor pathway against ferroptosis.

In addition to GPX4, several GPX4-independent anti-ferroptosis systems have been identified. Ferroptosis suppressor protein 1 (FSP1) acts as a GSH-independent ferroptosis suppressor by reducing coenzyme Q10 (CoQ10) to its antioxidant form at the plasma membrane [98,99]. Moreover, dihydroorotate dehydrogenase (DHODH) provides another CoQ-linked defense system at the mitochondrial inner membrane, suppressing mitochondrial lipid peroxidation and ferroptosis [100]. Recent studies have identified several lipid-soluble antioxidant pathways that also repress ferroptosis. GTP cyclohydrolase 1 (GCH1)-mediated tetrahydrobiopterin (BH4) synthesis counteracts ferroptosis by limiting lipid peroxidation [101,102]. In addition, vitamin K reduction mediated by vitamin K epoxide reductase complex subunit 1-like protein 1 (VKORC1L1) or FSP1 has been identified as another ferroptosis surveillance pathway associated with tumor suppression [103,104,105]. The intermediate metabolite of cholesterol metabolism, 7-dehydrocholesterol, also possesses anti-ferroptosis effects, and it is metabolized by sterol C5-desaturase (SC5D) [106]. In addition, iPLA2β, encoded by calcium-independent phospholipase A2 (*PLA2G6*), can mediate lipid detoxification and control p53-driven ferroptosis independently of GPX4 [107,108] (Figure 2, right panel).

## 4. HBV-Mediated Lipid Metabolism Remodeling and Ferroptosis Susceptibility

HBV infection profoundly rewires hepatic lipid metabolism. This is particularly relevant to ferroptosis because the ferroptotic threshold is determined by lipid substrate availability. HBV and its encoded proteins, especially HBx, regulate lipogenesis, phospholipid synthesis, fatty acid uptake, and immune cell lipid signaling [109]. Thus, these metabolic alterations triggered by HBV may also reshape the susceptibility of hepatocytes, tumor cells, and immune cells to ferroptosis (Table 1).

### 4.1. Fatty Acid Metabolism

As a fundamental metabolic process, fatty acid metabolism is one of the most consistently altered metabolic programs during HBV infection. HBx interacts with liver X receptor α (LXRα), thereby promoting sterol regulatory element-binding protein 1 (SREBP1) and fatty acid synthase (FASN) expression and enhancing de novo fatty acid synthesis [23]. Furthermore, HBx has also been implicated in the activation of peroxisome proliferator-activated receptor gamma (PPARγ) signaling and subsequent lipogenesis during HBV-related HCC [110,111,112]. Given that the protective roles of de novo lipogenesis-associated genes, such as FASN and stearoyl-CoA desaturase 1 (SCD1), in ferroptosis resistance have been well established, a functional link between HBx-mediated de novo lipogenesis and altered ferroptosis sensitivity is plausible [113,114]. Indeed, Zhang et al. showed that targeting hepatitis B X-interacting protein (HBXIP)-associated SCD1 upregulation enhances sorafenib-induced ferroptosis, thereby suggesting a potential therapeutic strategy for HCC [30]. Although the authors did not explicitly examine this mechanism in the context of HBV-related HCC, their findings in HBV genome-containing Hep3B cells provide indirect support for this possibility.

Moreover, HBx-induced dysregulation of fatty acid metabolism, which contributes to liver cancer development, has been widely observed in cell and mouse models [115]. Furthermore, the cooperative effect of ethanol and HBV in triggering lipid remodeling has been well documented in vivo and in vitro. For instance, HBx-mediated loss of glutamic pyruvate transaminase 2 (GPT2) promotes HCC progression by modulating alcohol dehydrogenase 1A (ADH1A) and lipid accumulation. In addition, activating transcription factor 4 (ATF4)-induced expression of lysophospholipase A2 (LYPLA2) has been observed in HBx-transgenic (HBx-Tg) mice and HBV-related HCC [24,116]. Chronic ethanol intake also amplifies the HBV-associated abnormal lipid signaling through the HBx-mediated arachidonic acid (C20:4) pathway and activates regulatory T cells (Tregs), indirectly suggesting that arachidonic acid may modulate the liver microenvironment independent of its pro-ferroptotic effects [117]. Finally, HBx also regulates fatty acid uptake: HBx-induced long-chain fatty acid transport protein 2 (FATP2) upregulation drives lipid accumulation and hepatic steatosis [17]. Taken together, although these studies do not always directly measure the role of HBV/HBx-induced fatty acid metabolism remodeling in ferroptosis, they suggest that HBV creates a lipid environment in which ferroptosis sensitivity may be altered during the progression of HCC.

### 4.2. Phospholipid Metabolism

Phospholipid metabolism represents a more direct connection between HBV replication and ferroptosis. HBV replication depends on host phospholipid synthesis. Enhanced phospholipid synthesis has been reported to be critical for HBV replication [118]. This finding suggests that HBV remodels host membranes for viral replication, and it is unsurprising that this same increase in phospholipid biosynthesis may also expand the pool of oxidizable substrates required for ferroptosis. For example, LPCAT3 has been defined as a potential biomarker for HBV replication and chronic HBV progression [119]. Given that LPCAT3 promotes ferroptosis by cooperating with ACSL4 to synthesize PUFA-PLs, its upregulation may indicate enhanced ferroptosis susceptibility in HBV-infected hepatic cells [120]. Chen et al. reported that ACSL4 was significantly upregulated in HBV-related HCC liver tissues and that ACSL4 plays a role in HBV-HCC progression by modulating bile acid-mediated M2 macrophage polarization [26]. This study is consistent with a previous report showing that HBx upregulates ACSL4 through miR-205 suppression, providing further indirect evidence for the link between phospholipid metabolism remodeling during HBV infection and potential ferroptosis susceptibility [27].

Phospholipid alterations also shape antiviral immunity. HBV-driven lipid alterations can contribute to natural killer T (NKT) cell-dependent protective immunity during early HBV infection [121]. Conversely, distinct lipid alterations in HCC are associated with impaired T cell-dependent antitumor immunity [122]. Since different T-cell states exhibit distinct phospholipid profiles and ACSL4-dependent ferroptosis sensitivity [123], HBV-driven phospholipid remodeling may affect not only hepatocytes but also immune cell survival and function. Therefore, HBV-mediated phospholipid remodeling may have dual consequences: increased phospholipid synthesis supports viral replication but may also lower the threshold for ferroptosis.

### 4.3. Other Lipid Metabolic Pathways

Beyond fatty acids and phospholipids, other lipid classes also influence HBV infection and ferroptosis sensitivity. For instance, acyl-CoA:cholesterol acyltransferase/sterol O-acyltransferase (ACAT/SOAT) has been proposed as a dual viral and T-cell metabolic checkpoint [124]. Given the established role of ACAT/SOAT in defending against ferroptosis, it is tempting to speculate that the enhancement of anti-HBV and anti-HCC responses induced by ACAT/SOAT inhibition may be partly mediated by increased ferroptotic cell death [125]. Furthermore, long-chain acylcarnitines, metabolites reported to have ferroptosis-inducing capacity, can also induce senescence of invariant natural killer T (iNKT) cells in HBV-related HCC [126,127]. Interestingly, extracellular vesicle-mediated lipid regulation also connects HBV-positive tumor cells with ferroptosis in the microenvironment. Exosomal miR-142-3p secreted by HBV-positive HepG2.2.15 cells can promote ferroptosis of M1-type macrophages through solute carrier family 3 member 2 (SLC3A2), thereby facilitating HCC progression [18,19]. Similarly, HBV-infected hepatocyte-derived exosomes can promote liver fibrosis through transferrin receptor (TFRC)-mediated ferroptosis induction [13]. These studies provide direct evidence that HBV coordinates cellular communication through exosomal cargo to modulate ferroptosis in the liver microenvironment.

Several recent studies further connect HBV-related HCC with ferroptosis regulators. HCC cells with high S100P expression exhibit a ferroptosis-resistant phenotype through lipid metabolic rewiring, thereby facilitating HCC development [31]. While this study did not directly dissect the upstream regulatory role of the virus, its findings in an HBV genome-containing Hep3B cell line further support the extrapolated notion that HBV-associated contexts promote HCC progression via lipid metabolic remodeling. In contrast to this indirect correlation, direct evidence demonstrates that multiple inositol polyphosphate phosphatase 1 (MINPP1) promotes ferroptosis via cathepsin B (CTSB) deubiquitination exclusively in HBV-positive HCC, revealing a virus-specific pathway inactive in HBV-negative cells [20]. Additionally, elevated HBx expression in HBV-associated HCC inhibits erastin-induced ferroptosis through upregulation of the ferroptosis suppressor sirtuin 1 (SIRT1) [21].

Finally, non-classical lipid antioxidant systems may be relevant to HBV-related disease. Although the specific roles of the FSP1-CoQ10, DHODH-CoQ10, FSP1/VKORC1L1-vitamin K, and GCH1-BH4 pathways in HBV infection remain incompletely defined, these systems may determine whether HBV-driven lipid remodeling culminates in ferroptosis or is buffered by compensatory antioxidant networks. Therefore, future studies should examine how HBV and HBx regulate these lipid antioxidant systems in hepatocytes and immune cells.

## 5. Reconstitution of Liver Microenvironment by HBV-Ferroptosis Crosstalk

**Figure 3:**
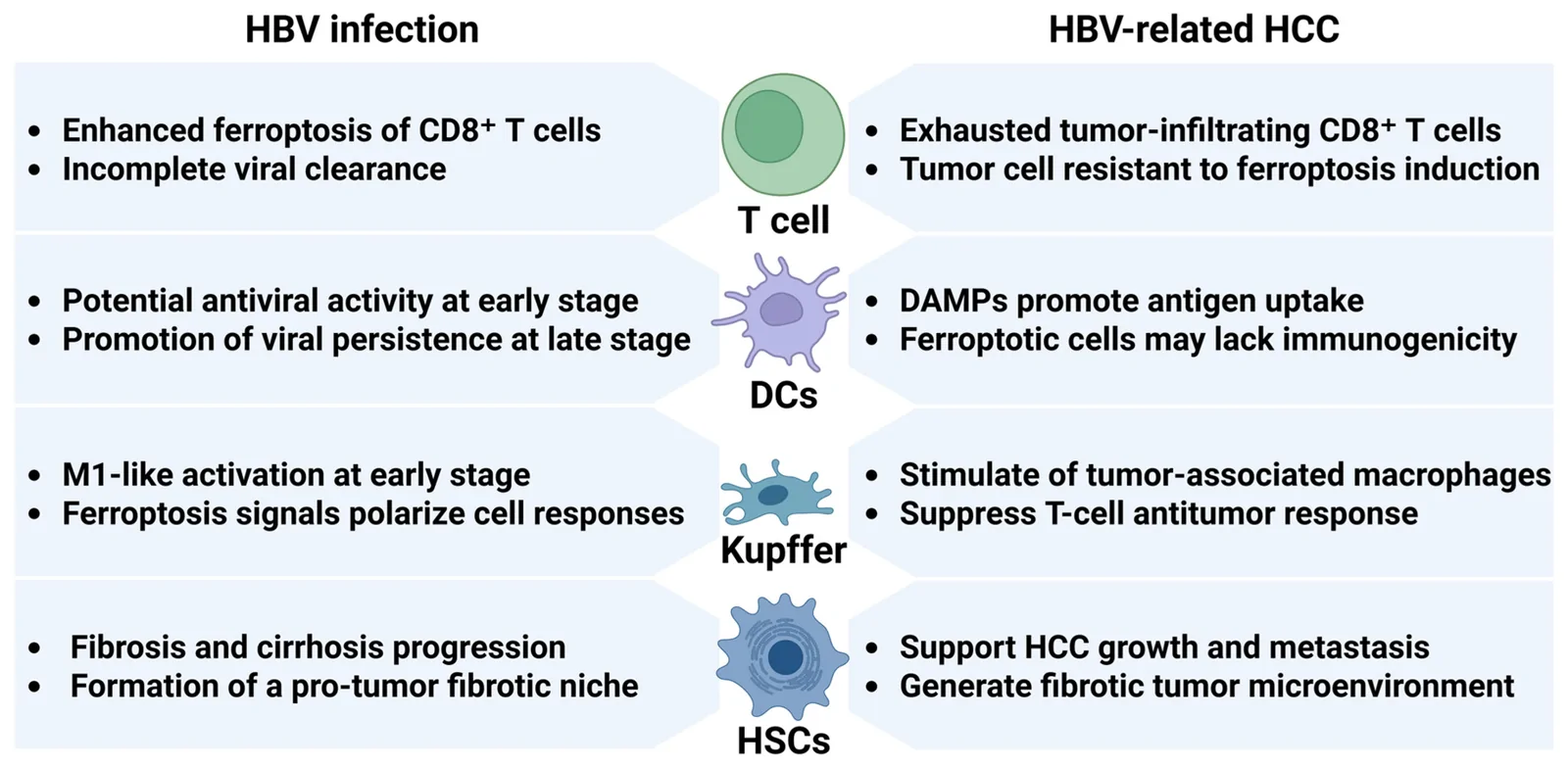
Cell type-specific roles of ferroptosis-related responses during HBV infection and HBV-related HCC. This schematic summarizes how ferroptosis-associated events differentially regulate liver immune and stromal cells during HBV infection and HBV-related hepatocellular carcinoma. Abbreviations: HBV, hepatitis B virus; HCC, hepatocellular carcinoma; CD8^+^ T cells, cytotoxic T lymphocytes; DCs, dendritic cells; HSCs, hepatic stellate cells; DAMPs, damage-associated molecular patterns. Created in BioRender. Yang, X. (2026) https://BioRender.com/zetesmq.

HBV infection creates several conditions that may favor ferroptosis-related microenvironmental remodeling: oxidative stress, altered lipid metabolism, and DAMP release. Kuo et al. reported that clearance of HSCs via the induction of ferroptosis efficiently limits HBV-driven fibrosis, providing direct evidence that ferroptosis participates in HBV-driven fibrogenic remodeling [14]. Moreover, HBx-mediated BAP1 ubiquitination enhances SLC7A11 expression and thereby promotes ferroptosis resistance, which may in turn contribute to the formation of a pro-inflammatory microenvironment in HBV-related HCC [22]. Here, we briefly summarize the current evidence regarding potential HBV-ferroptosis crosstalk across several immune cell subsets (Figure 3).

### 5.1. Adaptive Immune Cells

Adaptive immunity, especially HBV-specific CD8^+^ T-cell immunity, is essential for viral control. Impaired T-cell function and immune exhaustion have been characterized as typical signatures during chronic HBV infection at both molecular and cellular levels. For example, lipid metabolites released by hepatocytes or T cells facilitate chronic HBV infection and hinder the antiviral immune response [64]. Toll-like receptor 2 (TLR2) contributes to CD8^+^ T-cell exhaustion in HBV infection [128]. Crucially, direct clinical evidence demonstrates that the ferroptosis of immune cells contributes to immune failure during HBV infection, a process dictated by CD36-mediated lipid transport and GPX4 downregulation. Thus, targeting ferroptosis has been reported to restore antiviral CD8^+^ T-cell activity [11,129]. Extrapolating from these viral-specific contexts, an intact anti-ferroptotic defense system appears to be important for maintaining T-cell longevity and anti-infectious capacity. For instance, maintaining GPX4 activity and follicular helper T (T_fh_) cell survival by selenium supplementation can enhance the vaccine responses of young adults, indicating the importance of immune reconstitution in antiviral immunity [130]. Conversely, CD4^+^ T cells lacking murine *Gpx4* fail to expand and to protect the host from acute virus or parasite infections, while the AKT-GSK3β axis can promote the longevity of virus-specific memory CD4^+^ T cells by preventing ferroptosis [131,132]. These two studies suggest that an intact ferroptosis defense system is important for T-cell-mediated anti-infectious activities.

At the tumor cell level, CD8^+^ T cells can induce ferroptosis in tumor cells during immunotherapy by releasing IFN-γ, which suppresses system Xc^−^ components such as SLC7A11 and SLC3A2 and increases tumor cell lipid peroxidation [133]. However, chronic HBV infection often induces CD8^+^ T-cell exhaustion and metabolic dysfunction, which may weaken this antitumor capacity in HBV-related HCC. To address this issue, Hou and colleagues found that the application of bevacizumab, a recombinant humanized monoclonal antibody against VEGFA, induces ferroptosis and enhances CD8^+^ T-cell immune activity in liver cancer via the modulation of histone acetyltransferases [134]. Beyond epigenetic modulation, blocking ferroptosis in CD8^+^ T cells by either the iron chelator deferoxamine or ferritin heavy chain (FTH1) knockdown effectively restored their antitumor activity in HCC mouse models [135]. Collectively, while a unified molecular axis integrating these pathways under explicit HBV pressure remains to be directly validated, these findings imply that a bidirectional ferroptotic strategy—safeguarding CD8^+^ T cells while concurrently driving HCC cell death—may be important for successful therapy.

### 5.2. Dendritic Cells

Dendritic cells (DCs) are central antigen-presenting cells that bridge innate and adaptive immunity. The activities of DCs are jointly determined by their microenvironment and external stimuli. During HBV infection, human peripheral blood mononuclear cell-derived DCs can inhibit HBV replication in HepG2.2.15 cells, indicating their potential antiviral activity [136]. However, HBV can impair the protective immune crosstalk between DCs and other immune cells, thereby weakening antiviral immunity [137]. Under hypoxic conditions, type 2 conventional dendritic cells (cDC2) are highly enriched in HCC, indicating that specific DCs also play an immunosuppressive role in HCC progression. Moreover, direct experimental evidence indicates that a lipid-rich microenvironment upregulates T-cell immunoglobulin and mucin domain-containing protein 3 (TIM-3) in DCs, thereby triggering ferroptosis in DCs and promoting liver tumor progression [138].

Nevertheless, how ferroptosis affects DC function still remains unclear. On the one hand, ferroptotic hepatocytes or tumor cells can release oxidized lipids, HMGB1, and other DAMPs that promote antigen uptake and immune activation. Consistently, Efimova and colleagues found that ferroptotic cells are immunogenic and can sufficiently promote the maturation of DCs [139]. On the other hand, excessive lipid peroxidation may impair DC maturation or induce tolerogenic phenotypes. A recent study indicated that ferroptotic cancer cells lack immunogenicity, which impedes antigen presentation to DCs and hinders the therapeutic applications of ferroptosis [140]. Therefore, in chronic HBV infection and HBV-related HCC progression, excessive ferroptosis-derived signals may inhibit antigen presentation and contribute to dysfunctional DC responses [141,142,143]. Although direct studies on HBV-DC-ferroptosis crosstalk remain limited, clarifying the mechanisms involved may provide important opportunities for antiviral and antitumor intervention.

### 5.3. Kupffer Cells

Kupffer cells (KCs) are liver-resident macrophages that sense pathogens, clear dying cells, and orchestrate inflammatory responses. HBV particles can preferentially induce KCs to produce TGF-β1 rather than pro-inflammatory cytokines, favoring an immunosuppressive and fibrogenic milieu [144]. TLR2 enables KCs to support CD8^+^ T-cell exhaustion in HBV models [128]. Zhang and colleagues found that NCF1 causes iron overload and triggered ferroptosis in resident KCs. This KC ferroptosis impairs their self-renewal and immune homeostasis, thereby accelerating metabolic dysfunction-associated steatohepatitis (MASH) progression [145]. Ferroptosis-related signals may polarize KC responses. For example, HMGB1 is implicated in liver disease progression and can be induced by HBx, promoting angiogenesis through signal transducer and activator of transcription 3 (STAT3)-mediated NF-κB signaling [25]. In addition, viral dsDNA-mediated activation of the cyclic GMP-AMP synthase-stimulator of interferon genes protein (cGAS-STING) pathway can promote the M1 polarization of macrophages and antiviral activity against HBV [146]. Therefore, ferroptotic damage in the HBV-infected liver may act as a second signal that reshapes KC fate.

### 5.4. Hepatic Stellate Cells

HSCs are the principal fibrogenic cells in the liver and play a central role in the progression from fibrosis to hepatocellular carcinoma. Zhang et al. reported that exosomal miR-222 derived from HBV-infected hepatocytes promoted liver fibrosis by suppressing TFRC-dependent ferroptosis in HSCs [13]. This study is consistent with several recent reports revealing that the inhibition of HSC ferroptosis promotes liver fibrosis through the myosin 9/lipocalin 2 (MYH9/LCN2) axis, YTH domain-containing family protein 1 (YTHDF1)-mediated m6A modification, or cholesterol metabolism [28,147,148]. Furthermore, the DAMPs released from dead cells could also modulate HSC activity. An and colleagues found that mitochondria-derived danger signals, primarily mtDNA, could activate HSCs and thereby promote liver fibrosis [9]. Lipid peroxidation-derived aldehydes, such as 4-HNE, can also function as a profibrogenic stimulus in activated human HSCs [10]. Since HSC activation plays a critical role during HBV-driven fibrosis, triggering HSC ferroptosis could be an efficient strategy against HBV-driven fibrosis. Indeed, chrysophanol, a naturally occurring organic compound belonging to the anthraquinone family, enhanceS HSC ferroptosis to mitigate the HBV-driven fibrogenic process, an effect which can be blocked by an iron chelator [14,15]. By leveraging a similar therapeutic concept, dihydroartemisinin (an antimalarial agent) can likewise alleviate hepatic fibrosis through the induction of HSC ferroptosis [29]. Although the latter study was conducted in a general liver injury model rather than an explicit viral context, its findings corroborate the broader applicability of this strategy.

## 6. Conclusions and Perspective

HBV-driven lipid metabolic remodeling links viral persistence, chronic inflammation, immune dysfunction, and hepatocarcinogenesis to ferroptosis-regulated cell fate. By reshaping fatty acid profiles, phospholipid composition, and lipid-soluble antioxidant systems, HBV may either sensitize hepatocytes to peroxidative injury or promote ferroptosis resistance in transformed cells. However, this “double-edged sword” model should be regarded as a conceptual framework, as direct HBV-specific evidence remains incomplete.Biomarker-driven stratification will be essential for safe and effective clinical translation. Candidate biomarkers may include ferroptosis-related regulators, lipidomic features, and oxidative stress markers, integrated with HBV-specific clinical variables such as viral load, fibrosis stage, and treatment status. From a clinical perspective, defining the temporal and disease stage-specific windows in which ferroptosis operates may be particularly important in HBV-related liver disease. If the critical window during which HBV-driven ferroptosis contributes to chronic liver injury, inflammation, fibrosis, and malignant transformation can be identified, ferroptosis inhibition may help attenuate inflammatory and fibrotic remodeling and reduce the risk of HBV-related hepatocarcinogenesis. In contrast, once HCC has developed, therapeutic induction of ferroptosis may represent a valuable strategy to eliminate tumor cells, especially when combined with conventional treatment modalities or immune checkpoint inhibitors. Therefore, a stage-specific approach to ferroptosis modulation—blocking detrimental ferroptosis during chronic HBV-related liver injury while promoting ferroptosis in established tumors—may offer important opportunities for both the prevention and treatment of HBV-related HCC.

## Figures and Tables

**Table 1:** Summary of evidence connecting HBV-driven lipid metabolic regulators with ferroptosis during HBV-related liver disease progression.

Disease Context	Ferroptosis- or Lipid-Related Regulator	Experimental Model and Cell Type	HBV/HBx-Related Trigger	Evidence Level	Main Finding and Ferroptosis Relevance	Ref.
HBV infection	CD36; GPX4	chronic HBV infection mouse model; CD8^+^ T cells	HBV-specific CD8^+^ T cells	direct	In chronic HBV infection, HBV-specific CD8^+^ T cells exhibit increased CD36 expression and reduced GPX4 expression, which is associated with enhanced ferroptosis susceptibility.	[11]
HBV-related acute liver failure	SLC7A11	D-GalN-induced acute liver failure model; HBx-transgenic mice	HBx-driven SLC7A11 repression	direct	HBx aggravated D-GalN-induced acute liver failure in HBx-transgenic mice by repressing SLC7A11 expression, thereby promoting ferroptosis.	[12]
HBV-related liver fibrosis	miR-222	LO2 cells infected with HBV; HSC line LX-2	exosome secreted from HBV-infected hepatocytes	direct	Exosomal miR-222 released from HBV-infected hepatocytes promotes liver fibrosis by inhibiting TFRC-dependent ferroptosis in HSCs, thereby contributing to liver fibrosis.	[13]
	GPX4; SLC7A11	rat HSC line HSC-T6	HBx-induced HSC activation	direct	Chrysophanol induces ferroptosis in HBx-activated HSCs by suppressing GPX4 and SLC7A11, thereby inhibiting fibrosis.	[14,15]
	TRIM37	HSC line LX-2 infected with HBV	HBV-driven ROS generation	indirect	HBV/HBx increased ROS generation and TRIM37 expression, implying a indirect link to ferroptosis-related signaling.	[16]
	FATP2	HepG2.2.15 cell line	HBx-driven FATP2 expression	indirect	HBx-induced FATP2 upregulation promoted lipid accumulation, which may contribute to a ferroptosis-prone metabolic state.	[17]
HBV-related HCC	miR-142-3p; SLC3A2	DEN (diethylnitrosamine)-induced HCC model; HepG2.2.15 cell line	exosome secreted from HBV-infected HCC cells	direct	Exosomal miR-142-3p derived from HBV-infected HCC cells downregulates SLC3A2 in M1 macrophages, thereby promoting macrophage ferroptosis and facilitating HCC progression.	[18,19]
	MINPP1; CTSB	HBV-related HCC model; Hep3B cell line	HBV-positive HCC context	direct	MINPP1 modulates the deubiquitination of CTSB, thereby promoting ferroptosis selectively in HBV-positive, but not HBV-negative, HCC cells.	[20]
	SIRT1	HepG2.2.15 cell line	HBx-induced SIRT1 expression	direct	In HBV-positive HCC cells, HBx upregulates SIRT1, thereby suppressing ferroptosis.	[21]
	BAP1; SLC7A11	HepG2.2.15 and Hep3B cell lines	HBx reduces BAP1 stability	direct	HBx reduced BAP1 protein stability, leading to increased SLC7A11 expression and suppressed ferroptosis in HCC cells.	[22]
	LXRα; SREBP-1; FASN	HBx-transgenic mice	HBx-driven LXRα activation	indirect	HBx activates the LXRα–SREBP1–FASN axis and promotes de novo lipogenesis, which may contribute to lipid remodeling associated with ferroptosis susceptibility in HBV-related HCC.	[23]
	ATF4; LYPLA2	HBx-transgenic mice; HepG2.2.15 cell line	HBV/HBx-driven ATF4 activation	indirect	ATF4/LYPLA2 signaling promotes lipid remodeling in HBV-related HCC, and ATF4 is a ferroptosis-suppressive factor, suggesting a possible indirect link to ferroptosis resistance.	[24]
	HMGB1	xenograft model	HBx-driven HMGB1 expression	indirect	HBx upregulated HMGB1 expression in HBV-related HCC. This finding may indirectly connect HBx-driven tumor progression with ferroptosis-associated pathways.	[25]
	ACSL4	HSC line LX-2 infected with HBV; macrophage	ACSL4 upregulation in HBV-related HCC	indirect	ACSL4 was associated with M2 macrophage polarization and progression of HBV-related HCC. ACSL4 is a ferroptosis-promoting factor.	[26]
	miR-205; ACSL4	HBx-transgenic mice model	HBx-mediated miRNA regulation	indirect	HBx suppresses miR-205, leading to ACSL4 upregulation, which may increase ferroptosis susceptibility.	[27]
Liver fibrosis (non-HBV specific)	GPX4	HSC lines, LX-2 and TWNT-1	N/A	extrapolated	Statins inhibit hepatic stellate cell activation by targeting the GPX4-mediated anti-ferroptotic pathway.	[28]
	NCOA4	Primary HSCs	N/A	extrapolated	Dihydroartemisinin induces ferroptosis in hepatic stellate cells, thereby attenuating liver fibrosis.	[29]
HCC (non-HBV specific)	HBXIP; SCD1	xenograft model; Hep3B cell line	HBXIP-mediated SCD1 induction	indirect	HBXIP induced the expression of SCD1, resulting in the suppression of ferroptosis and HCC progression.	[30]
	S100P; ACC1	DEN-induced HCC model; Hep3B cell line	N/A	extrapolated	Loss of S100P increases acetyl-CoA carboxylase 1 (ACC1) expression and promotes ferroptosis in HBV-positive HCC cells.	[31]

Note: Evidence level was categorized as direct, indirect, or extrapolated. Direct evidence indicates that HBV/HBx-related models experimentally demonstrated a mechanistic link between lipid metabolic regulation and ferroptosis-related phenotypes. Indirect evidence indicates that HBV/HBx-related studies identified lipid remodeling, oxidative stress, or disease-promoting effects without directly validated relevance to ferroptosis. Extrapolated evidence indicates mechanisms inferred from non-HBV HCC and other liver disease models.

## Data Availability

No new data were created or analyzed in this study. Data sharing is not applicable to this article.

## References

[1] Hsu Y.-C., Huang D.Q., Nguyen M.H. (2023). Global burden of hepatitis B virus: Current status, missed opportunities and a call for action. Nat. Rev. Gastroenterol. Hepatol..

[2] Banach M. (2022). Global, regional, and national burden of hepatitis B, 1990–2019: A systematic analysis for the Global Burden of Disease Study 2019. GBD 2019 Hepatitis B Collaborator. Lancet Gastroenterol. Hepatol..

[3] Chen C.-J., Yang H.-I., Su J., Jen C.-L., You S.-L., Lu S.-N., Huang G.-T., Iloeje U.H., Group R.-H.S. (2006). Risk of hepatocellular carcinoma across a biological gradient of serum hepatitis B virus DNA level. JAMA.

[4] Tseng T.C., Liu C.J., Yang H.C., Su T.H., Wang C.C., Chen C.L., Kuo S.F.T., Liu C.H., Chen P.J., Chen D.S. (2012). High levels of hepatitis B surface antigen increase risk of hepatocellular carcinoma in patients with low HBV load. Gastroenterology.

[5] Choi W.-M., Yip T.C.-F., Kim W.R., Yee L.J., Brooks-Rooney C., Curteis T., Clark L.J., Jafry Z., Chen C.-H., Chen C.-Y. (2024). Chronic hepatitis B baseline viral load and on-treatment liver cancer risk: A multinational cohort study of HBeAg-positive patients. Hepatology.

[6] Vucur M., Kondylis V., Broz P., Luedde T. (2025). Regulated necrosis at the crossroads of liver inflammation and cancer development. Nat. Rev. Gastroenterol. Hepatol..

[7] Hammerich L., Tacke F. (2023). Hepatic inflammatory responses in liver fibrosis. Nat. Rev. Gastroenterol. Hepatol..

[8] Mederacke I., Filliol A., Affo S., Nair A., Hernandez C., Sun Q., Hamberger F., Brundu F., Chen Y., Ravichandra A. (2022). The purinergic P2Y14 receptor links hepatocyte death to hepatic stellate cell activation and fibrogenesis in the liver. Sci. Transl. Med..

[9] An P., Wei L.-L., Zhao S., Sverdlov D.Y., Vaid K.A., Miyamoto M., Kuramitsu K., Lai M., Popov Y.V. (2020). Hepatocyte mitochondria-derived danger signals directly activate hepatic stellate cells and drive progression of liver fibrosis. Nat. Commun..

[10] Zamara E., Novo E., Marra F., Gentilini A., Romanelli R.G., Caligiuri A., Robino G., Tamagno E., Aragno M., Danni O. (2004). 4-Hydroxynonenal as a selective pro-fibrogenic stimulus for activated human hepatic stellate cells. J. Hepatol..

[11] Li H., Xiao S., Huo C., Yang S., Wang J., Lan X., Li M., Shi L., Zhuo L., Zhang J. (2025). Targeting ferroptosis restores the antiviral activity of CD8+ T cells during chronic HBV infection. Cell. Mol. Gastroenterol. Hepatol..

[12] Liu G.-Z., Xu X.-W., Tao S.-H., Gao M.-J., Hou Z.-H. (2021). HBx facilitates ferroptosis in acute liver failure via EZH2 mediated SLC7A11 suppression. J. Biomed. Sci..

[13] Zhang Q., Qu Y., Zhang Q., Li F., Li B., Li Z., Dong Y., Lu L., Cai X. (2023). Exosomes derived from hepatitis B virus-infected hepatocytes promote liver fibrosis via miR-222/TFRC axis. Cell Biol. Toxicol..

[14] Kuo C.-Y., Chiu V., Hsieh P.-C., Huang C.-Y., Huang S.J., Tzeng I.-S., Tsai F.-M., Chen M.-L., Liu C.-T., Chen Y.-R. (2020). Chrysophanol attenuates hepatitis B virus X protein-induced hepatic stellate cell fibrosis by regulating endoplasmic reticulum stress and ferroptosis. J. Pharmacol. Sci..

[15] Tseng H.-F., Hsieh P.-C., Lan C.-C., Chiu V., Kuo C.-Y. (2022). Desferal (Deferoxamine) Targets Ferroptosis Triggered by Chrysophanol in Hepatic B Virus X Protein-Induced Hepatic Stellate Cell Activation. Curr. Top. Nutraceutical Res..

[16] Xie H., Xie D., Zhang J., Jin W., Li Y., Yao J., Pan Z., Xie D. (2020). ROS/NF-κB signaling pathway-mediated transcriptional activation of TRIM37 promotes HBV-associated hepatic fibrosis. Mol. Ther. Nucleic Acids.

[17] Lu Y., Yang X., Kuang Q., Wu Y., Tan X., Lan J., Qiang Z., Feng T. (2023). HBx induced upregulation of FATP2 promotes the development of hepatic lipid accumulation. Exp. Cell Res..

[18] Hu Z., Yin Y., Jiang J., Yan C., Wang Y., Wang D., Li L. (2022). Exosomal miR-142-3p secreted by hepatitis B virus (HBV)-hepatocellular carcinoma (HCC) cells promotes ferroptosis of M1-type macrophages through SLC3A2 and the mechanism of HCC progression. J. Gastrointest. Oncol..

[19] Hu Z., Zhang H., Liu W., Yin Y., Jiang J., Yan C., Wang Y., Li L. (2022). Mechanism of HBV-positive liver cancer cell exosomal miR-142-3p by inducing ferroptosis of M1 macrophages to promote liver cancer progression. Transl. Cancer Res..

[20] Chen W., Song Y., Wang L., Xiong F., Zhang S. (2025). MINPP1 promotes ferroptosis in HBV-related hepatocellular carcinoma by regulating CTSB K33-linked deubiquitination via ZRANB1. Biol. Direct.

[21] Wang N., Duan Z., Chen Z., Liu C., Huang Y., Li Z. (2026). HBx Inhibits the Ferroptosis in HBV-Associated Hepatocellular Carcinoma Through Upregulation of SIRT1. J. Appl. Toxicol..

[22] Li D., Li B., Zhao X., Dong R., Zhang M., Yin J., Peng Y., Zhao Z., Ni B. HBx-Mediated BAP1 Ubiquitination Promotes Hcc Pathogenesis through Ferroptosis Resistance by Activating cGAS-STING Signaling. https://ssrn.com/abstract=7146222.

[23] Kim K., Kim K.H., Kim H.H., Cheong J. (2008). Hepatitis B virus X protein induces lipogenic transcription factor SREBP1 and fatty acid synthase through the activation of nuclear receptor LXRα. Biochem. J..

[24] Zhou H., Ba J., Xiao C., Liu H., Jiang J., Guo Y., Wu B. (2025). Alcohol activates ATF4/LPLA2-mediated BMP metabolism to enhance HBV-induced hepatocellular carcinogenesis. J. Hepatol..

[25] Zhang Y., Ren H., Li J., Xue R., Liu H., Zhu Z., Pan C., Lin Y., Hu A., Gou P. (2021). Elevated HMGB1 expression induced by hepatitis B virus X protein promotes epithelial-mesenchymal transition and angiogenesis through STAT3/miR-34a/NF-κB in primary liver cancer. Am. J. Cancer Res..

[26] Chen W., Xu H., Guo L., Zheng F., Yao J., Wang L. (2024). Role of ACSL4 in modulating farnesoid X receptor expression and M2 macrophage polarization in HBV-induced hepatocellular carcinoma. MedComm.

[27] Cui M., Xiao Z., Sun B., Wang Y., Zheng M., Ye L., Zhang X. (2014). Involvement of cholesterol in hepatitis B virus X protein-induced abnormal lipid metabolism of hepatoma cells via up-regulating miR-205-targeted ACSL4. Biochem. Biophys. Res. Commun..

[28] Kitsugi K., Noritake H., Matsumoto M., Hanaoka T., Umemura M., Yamashita M., Takatori S., Ito J., Ohta K., Chida T. (2023). Simvastatin inhibits hepatic stellate cells activation by regulating the ferroptosis signaling pathway. Biochim. Biophys. Acta (BBA)-Mol. Basis Dis..

[29] Zhang Z., Wang X., Wang Z., Zhang Z., Cao Y., Wei Z., Shao J., Chen A., Zhang F., Zheng S. (2021). Dihydroartemisinin alleviates hepatic fibrosis through inducing ferroptosis in hepatic stellate cells. BioFactors.

[30] Zhang L., Li X.-M., Shi X.-H., Ye K., Fu X.-L., Wang X., Guo S.-M., Ma J.-Q., Xu F.-F., Sun H.-M. (2023). Sorafenib triggers ferroptosis via inhibition of HBXIP/SCD axis in hepatocellular carcinoma. Acta Pharmacol. Sin..

[31] Yang M., Cui W., Lv X., Xiong G., Sun C., Xuan H., Ma W., Cui X., Cheng Y., Han L. (2025). S100P is a ferroptosis suppressor to facilitate hepatocellular carcinoma development by rewiring lipid metabolism. Nat. Commun..

[32] Tsukuda S., Watashi K. (2020). Hepatitis B virus biology and life cycle. Antivir. Res..

[33] Sureau C., Salisse J. (2013). A conformational heparan sulfate binding site essential to infectivity overlaps with the conserved hepatitis B virus a-determinant. Hepatology.

[34] Yan H., Zhong G., Xu G., He W., Jing Z., Gao Z., Huang Y., Qi Y., Peng B., Wang H. (2012). Sodium taurocholate cotransporting polypeptide is a functional receptor for human hepatitis B and D virus. eLife.

[35] Hong X., Schneider W.M., Rice C.M. (2025). Hepatitis B virus nucleocapsid assembly. J. Mol. Biol..

[36] Mendenhall M.A., Hong X., Hu J. (2023). Hepatitis B virus capsid: The core in productive entry and covalently closed circular DNA formation. Viruses.

[37] Nassal M. (2015). HBV cccDNA: Viral persistence reservoir and key obstacle for a cure of chronic hepatitis B. Gut.

[38] Decorsière A., Mueller H., Van Breugel P.C., Abdul F., Gerossier L., Beran R.K., Livingston C.M., Niu C., Fletcher S.P., Hantz O. (2016). Hepatitis B virus X protein identifies the Smc5/6 complex as a host restriction factor. Nature.

[39] Sekiba K., Otsuka M., Ohno M., Yamagami M., Kishikawa T., Suzuki T., Ishibashi R., Seimiya T., Tanaka E., Koike K. (2019). Inhibition of HBV transcription from cccDNA with nitazoxanide by targeting the HBx–DDB1 interaction. Cell. Mol. Gastroenterol. Hepatol..

[40] Cheng S.-T., Chen W.-X., Deng H.-J., He X., Zhang H., Tan M., Yu H.-B., Zhang Z.-Z., Ren J.-H., Yang M.-L. (2025). Glutamate dehydrogenase 1-dependent α-ketoglutarate promotes hepatitis B virus transcription by modulating histone methylations on the covalently closed circular DNA minichromosome. Clin. Mol. Hepatol..

[41] Zhao Q., Chang J., Rijnbrand R., Lam A.M., Sofia M.J., Cuconati A., Guo J.-T. (2022). Pregenomic RNA launch hepatitis B virus replication system facilitates the mechanistic study of antiviral agents and drug-resistant variants on covalently closed circular DNA synthesis. J. Virol..

[42] Zhou L., Liu C.-H., Lv D., Sample K.M., Rojas Á., Zhang Y., Qiu H., He L., Zheng L., Chen L. (2025). Halting hepatocellular carcinoma: Identifying intercellular crosstalk in HBV-driven disease. Cell Rep..

[43] Sivasudhan E., Blake N., Lu Z., Meng J., Rong R. (2022). Hepatitis B viral protein HBx and the molecular mechanisms modulating the hallmarks of hepatocellular carcinoma: A comprehensive review. Cells.

[44] Chen L., Zhang C., Xue R., Liu M., Bai J., Bao J., Wang Y., Jiang N., Li Z., Wang W. (2024). Deep whole-genome analysis of 494 hepatocellular carcinomas. Nature.

[45] Svicher V., Salpini R., Piermatteo L., Carioti L., Battisti A., Colagrossi L., Scutari R., Surdo M., Cacciafesta V., Nuccitelli A. (2021). Whole exome HBV DNA integration is independent of the intrahepatic HBV reservoir in HBeAg-negative chronic hepatitis B. Gut.

[46] Rivière L., Quioc-Salomon B., Fallot G., Halgand B., Féray C., Buendia M.-A., Neuveut C. (2019). Hepatitis B virus replicating in hepatocellular carcinoma encodes HBx variants with preserved ability to antagonize restriction by Smc5/6. Antivir. Res..

[47] Sekiba K., Otsuka M., Funato K., Miyakawa Y., Tanaka E., Seimiya T., Yamagami M., Tsutsumi T., Okushin K., Miyakawa K. (2022). HBx-induced degradation of Smc5/6 complex impairs homologous recombination-mediated repair of damaged DNA. J. Hepatol..

[48] Yen C.-J., Lin Y.-J., Yen C.-S., Tsai H.-W., Tsai T.-F., Chang K.-Y., Huang W.-C., Lin P.-W., Chiang C.-W., Chang T.-T. (2012). Hepatitis B virus X protein upregulates mTOR signaling through IKKβ to increase cell proliferation and VEGF production in hepatocellular carcinoma. PLoS ONE.

[49] Zhang Y., Yan Q., Gong L., Xu H., Liu B., Fang X., Yu D., Li L., Wei T., Wang Y. (2021). C-terminal truncated HBx initiates hepatocarcinogenesis by downregulating TXNIP and reprogramming glucose metabolism. Oncogene.

[50] Li W., Li M., Liao D., Lu X., Gu X., Zhang Q., Zhang Z., Li H. (2016). Carboxyl-terminal truncated HBx contributes to invasion and metastasis via deregulating metastasis suppressors in hepatocellular carcinoma. Oncotarget.

[51] Li W., Duan X., Zhu C., Liu X., Jeyarajan A.J., Xu M., Tu Z., Sheng Q., Chen D., Zhu C. (2022). Hepatitis B and hepatitis C virus infection promote liver fibrogenesis through a TGF-β1–induced OCT4/Nanog pathway. J. Immunol..

[52] Li W., Yu X., Chen X., Wang Z., Yin M., Zhao Z., Zhu C. (2021). HBV induces liver fibrosis via the TGF-β1/miR-21-5p pathway. Exp. Ther. Med..

[53] Koyama Y., Brenner D.A. (2017). Liver inflammation and fibrosis. J. Clin. Investig..

[54] Yuan G., Chen B., Meng Y., Lu J., Shi X., Hu A., Hu Y., Wang D. (2021). Role of the CXCR3-mediated TLRs/MyD88 signaling pathway in promoting the development of hepatitis B into cirrhosis and liver cancer. Mol. Med. Rep..

[55] Wang X., Lin Y., Kemper T., Chen J., Yuan Z., Liu S., Zhu Y., Broering R., Lu M. (2020). AMPK and Akt/mTOR signalling pathways participate in glucose-mediated regulation of hepatitis B virus replication and cellular autophagy. Cell. Microbiol..

[56] Wang H., Zhang J. (2023). The glucose metabolic reprogramming in hepatitis B virus infection and hepatitis B virus associated diseases. J. Gastroenterol. Hepatol..

[57] Zhu Y., Zhao Y., Ning Z., Deng Y., Li B., Sun Y., Meng Z. (2024). Metabolic self-feeding in HBV-associated hepatocarcinoma centered on feedback between circulation lipids and the cellular MAPK/mTOR axis. Cell Commun. Signal..

[58] Li C., Zhao Z., Guo B., Xu D., Li S., Tang H., Jia B., Li F., Zhang J., Zou H. (2025). RAB1A promotes hepatitis B virus replication by enhancing PPARα-mediated viral transcription and inducing ULK1-mediated autophagy. Int. J. Biol. Macromol..

[59] Oh J., Baritugo K.-A.G., Kim J., Park G., Han K.J., Lee S., Sung G.-H. (2025). Metabolomic Profiling of Hepatitis B-Associated Liver Disease Progression: Chronic Hepatitis B, Cirrhosis, and Hepatocellular Carcinoma. Metabolites.

[60] Lan Y., Zhou Z., Huang Q., Yang X., Lu X., Jiang R., He Y., Yang X., Ye D., Chen Z. (2026). Integrated metabolomics analysis identifies distinct amino acid signatures in chronic hepatitis B patients with metabolic dysfunction-associated steatotic liver disease. Front. Cell. Infect. Microbiol..

[61] Zhou L., Xia S., Liu Y., Ji Q., Li L., Gao X., Guo X., Yi X., Chen F. (2023). A lipid metabolism–based prognostic risk model for HBV–related hepatocellular carcinoma. Lipids Health Dis..

[62] Zhou L., He R., Fang P., Li M., Yu H., Wang Q., Yu Y., Wang F., Zhang Y., Chen A. (2021). Hepatitis B virus rigs the cellular metabolome to avoid innate immune recognition. Nat. Commun..

[63] Yu Y., Wang Z., Yang A., Wang Y., Bao C., Zhuo L., Han Q., Zhao H., Zhang J. (2025). Chronic HBV infection impairs the glucose metabolism and effector function of NK cells via HBsAg/IL-15/mTOR axis. Cell Death Dis..

[64] Li H., Mu W., Zhao A., Xiao S., Huo C., Zhao H., Wang H., Li R., Han Q. (2025). Metabolic Reprogramming and CD 8+ T-Cell Exhaustion During Chronic HBV Infection. Liver Int..

[65] Dixon S.J., Lemberg K.M., Lamprecht M.R., Skouta R., Zaitsev E.M., Gleason C.E., Patel D.N., Bauer A.J., Cantley A.M., Yang W.S. (2012). Ferroptosis: An iron-dependent form of nonapoptotic cell death. Cell.

[66] Stockwell B.R. (2022). Ferroptosis turns 10: Emerging mechanisms, physiological functions, and therapeutic applications. Cell.

[67] Tang D., Chen X., Kang R., Kroemer G. (2021). Ferroptosis: Molecular mechanisms and health implications. Cell Res..

[68] Chen J., Li X., Ge C., Min J., Wang F. (2022). The multifaceted role of ferroptosis in liver disease. Cell Death Differ..

[69] Yang W.S., Kim K.J., Gaschler M.M., Patel M., Shchepinov M.S., Stockwell B.R. (2016). Peroxidation of polyunsaturated fatty acids by lipoxygenases drives ferroptosis. Proc. Natl. Acad. Sci. USA.

[70] Kagan V.E., Mao G., Qu F., Angeli J.P.F., Doll S., St Croix C., Dar H.H., Liu B., Tyurin V.A., Ritov V.B. (2017). Oxidized arachidonic and adrenic PEs navigate cells to ferroptosis. Nat. Chem. Biol..

[71] Doll S., Proneth B., Tyurina Y.Y., Panzilius E., Kobayashi S., Ingold I., Irmler M., Beckers J., Aichler M., Walch A. (2017). ACSL4 dictates ferroptosis sensitivity by shaping cellular lipid composition. Nat. Chem. Biol..

[72] Qiu B., Zandkarimi F., Bezjian C.T., Reznik E., Soni R.K., Gu W., Jiang X., Stockwell B.R. (2024). Phospholipids with two polyunsaturated fatty acyl tails promote ferroptosis. Cell.

[73] Samovich S.N., Mikulska-Ruminska K., Dar H.H., Tyurina Y.Y., Tyurin V.A., Souryavong A.B., Kapralov A.A., Amoscato A.A., Beharier O., Karumanchi S.A. (2024). Strikingly High Activity of 15-Lipoxygenase Towards Di-Polyunsaturated Arachidonoyl/Adrenoyl-Phosphatidylethanolamines Generates Peroxidation Signals of Ferroptotic Cell Death. Angew. Chem. Int. Ed..

[74] Magtanong L., Ko P.-J., To M., Cao J.Y., Forcina G.C., Tarangelo A., Ward C.C., Cho K., Patti G.J., Nomura D.K. (2019). Exogenous monounsaturated fatty acids promote a ferroptosis-resistant cell state. Cell Chem. Biol..

[75] Li Z., Hu Y., Zheng H., Li M., Liu Y., Feng R., Li X., Zhang S., Tang M., Yang M. (2024). LPCAT1-mediated membrane phospholipid remodelling promotes ferroptosis evasion and tumour growth. Nat. Cell Biol..

[76] Liang D., Feng Y., Zandkarimi F., Wang H., Zhang Z., Kim J., Cai Y., Gu W., Stockwell B.R., Jiang X. (2023). Ferroptosis surveillance independent of GPX4 and differentially regulated by sex hormones. Cell.

[77] Blomme A., Ford C.A., Mui E., Patel R., Ntala C., Jamieson L.E., Planque M., McGregor G.H., Peixoto P., Hervouet E. (2020). 2, 4-dienoyl-CoA reductase regulates lipid homeostasis in treatment-resistant prostate cancer. Nat. Commun..

[78] Nassar Z.D., Mah C.Y., Dehairs J., Burvenich I.J., Irani S., Centenera M.M., Helm M., Shrestha R.K., Moldovan M., Don A.S. (2020). Human DECR1 is an androgen-repressed survival factor that regulates PUFA oxidation to protect prostate tumor cells from ferroptosis. eLife.

[79] Cui W., Liu D., Gu W., Chu B. (2021). Peroxisome-driven ether-linked phospholipids biosynthesis is essential for ferroptosis. Cell Death Differ..

[80] Zou Y., Henry W.S., Ricq E.L., Graham E.T., Phadnis V.V., Maretich P., Paradkar S., Boehnke N., Deik A.A., Reinhardt F. (2020). Plasticity of ether lipids promotes ferroptosis susceptibility and evasion. Nature.

[81] Lange M., Wölk M., Li V.W., Doubravsky C.E., Hendricks J.M., Kato S., Otoki Y., Styler B., Johnson S.L., Harris C.A. (2025). FSP1-mediated lipid droplet quality control prevents neutral lipid peroxidation and ferroptosis. Nat. Cell Biol..

[82] Chu B., Kon N., Chen D., Li T., Liu T., Jiang L., Song S., Tavana O., Gu W. (2019). ALOX12 is required for p53-mediated tumour suppression through a distinct ferroptosis pathway. Nat. Cell Biol..

[83] Yang X., Wang Z., Samovich S.N., Kapralov A.A., Amoscato A.A., Tyurin V.A., Dar H.H., Li Z., Duan S., Kon N. (2024). PHLDA2-mediated phosphatidic acid peroxidation triggers a distinct ferroptotic response during tumor suppression. Cell Metab..

[84] Yang X., Gu W. (2024). PHLDA2 is critical for p53-mediated ferroptosis and tumor suppression. J. Mol. Cell Biol..

[85] Zou Y., Li H., Graham E.T., Deik A.A., Eaton J.K., Wang W., Sandoval-Gomez G., Clish C.B., Doench J.G., Schreiber S.L. (2020). Cytochrome P450 oxidoreductase contributes to phospholipid peroxidation in ferroptosis. Nat. Chem. Biol..

[86] Gao M., Monian P., Quadri N., Ramasamy R., Jiang X. (2015). Glutaminolysis and transferrin regulate ferroptosis. Mol. Cell.

[87] Gao M., Monian P., Pan Q., Zhang W., Xiang J., Jiang X. (2016). Ferroptosis is an autophagic cell death process. Cell Res..

[88] Hou W., Xie Y., Song X., Sun X., Lotze M.T., Zeh H.J., Kang R., Tang D. (2016). Autophagy promotes ferroptosis by degradation of ferritin. Autophagy.

[89] Mancias J.D., Wang X., Gygi S.P., Harper J.W., Kimmelman A.C. (2014). Quantitative proteomics identifies NCOA4 as the cargo receptor mediating ferritinophagy. Nature.

[90] Terzi E.M., Sviderskiy V.O., Alvarez S.W., Whiten G.C., Possemato R. (2021). Iron-sulfur cluster deficiency can be sensed by IRP2 and regulates iron homeostasis and sensitivity to ferroptosis independent of IRP1 and FBXL5. Sci. Adv..

[91] Wang L., Ouyang S., Li B., Wu H., Wang F. (2021). GSK-3β manipulates ferroptosis sensitivity by dominating iron homeostasis. Cell Death Discov..

[92] Anandhan A., Dodson M., Shakya A., Chen J., Liu P., Wei Y., Tan H., Wang Q., Jiang Z., Yang K. (2023). NRF2 controls iron homeostasis and ferroptosis through HERC2 and VAMP8. Sci. Adv..

[93] Zhang H.-L., Hu B.-X., Ye Z.-P., Li Z.-L., Liu S., Zhong W.-Q., Du T., Yang D., Mai J., Li L.-C. (2024). TRPML1 triggers ferroptosis defense and is a potential therapeutic target in AKT-hyperactivated cancer. Sci. Transl. Med..

[94] Jiang L., Kon N., Li T., Wang S.-J., Su T., Hibshoosh H., Baer R., Gu W. (2015). Ferroptosis as a p53-mediated activity during tumour suppression. Nature.

[95] Yang W.S., SriRamaratnam R., Welsch M.E., Shimada K., Skouta R., Viswanathan V.S., Cheah J.H., Clemons P.A., Shamji A.F., Clish C.B. (2014). Regulation of ferroptotic cancer cell death by GPX4. Cell.

[96] Dixon S.J., Patel D.N., Welsch M., Skouta R., Lee E.D., Hayano M., Thomas A.G., Gleason C.E., Tatonetti N.P., Slusher B.S. (2014). Pharmacological inhibition of cystine–glutamate exchange induces endoplasmic reticulum stress and ferroptosis. eLife.

[97] Friedmann Angeli J.P., Schneider M., Proneth B., Tyurina Y.Y., Tyurin V.A., Hammond V.J., Herbach N., Aichler M., Walch A., Eggenhofer E. (2014). Inactivation of the ferroptosis regulator Gpx4 triggers acute renal failure in mice. Nat. Cell Biol..

[98] Bersuker K., Hendricks J.M., Li Z., Magtanong L., Ford B., Tang P.H., Roberts M.A., Tong B., Maimone T.J., Zoncu R. (2019). The CoQ oxidoreductase FSP1 acts parallel to GPX4 to inhibit ferroptosis. Nature.

[99] Doll S., Freitas F.P., Shah R., Aldrovandi M., da Silva M.C., Ingold I., Goya Grocin A., Xavier da Silva T.N., Panzilius E., Scheel C.H. (2019). FSP1 is a glutathione-independent ferroptosis suppressor. Nature.

[100] Mao C., Liu X., Zhang Y., Lei G., Yan Y., Lee H., Koppula P., Wu S., Zhuang L., Fang B. (2021). DHODH-mediated ferroptosis defence is a targetable vulnerability in cancer. Nature.

[101] Kraft V.A., Bezjian C.T., Pfeiffer S., Ringelstetter L., Müller C., Zandkarimi F., Merl-Pham J., Bao X., Anastasov N., Kössl J. (2019). GTP cyclohydrolase 1/tetrahydrobiopterin counteract ferroptosis through lipid remodeling. ACS Cent. Sci..

[102] Soula M., Weber R.A., Zilka O., Alwaseem H., La K., Yen F., Molina H., Garcia-Bermudez J., Pratt D.A., Birsoy K. (2020). Metabolic determinants of cancer cell sensitivity to canonical ferroptosis inducers. Nat. Chem. Biol..

[103] Mishima E., Ito J., Wu Z., Nakamura T., Wahida A., Doll S., Tonnus W., Nepachalovich P., Eggenhofer E., Aldrovandi M. (2022). A non-canonical vitamin K cycle is a potent ferroptosis suppressor. Nature.

[104] Jin D.-Y., Chen X., Liu Y., Williams C.M., Pedersen L.C., Stafford D.W., Tie J.-K. (2023). A genome-wide CRISPR-Cas9 knockout screen identifies FSP1 as the warfarin-resistant vitamin K reductase. Nat. Commun..

[105] Yang X., Wang Z., Zandkarimi F., Liu Y., Duan S., Li Z., Kon N., Zhang Z., Jiang X., Stockwell B.R. (2023). Regulation of VKORC1L1 is critical for p53-mediated tumor suppression through vitamin K metabolism. Cell Metab..

[106] Freitas F.P., Alborzinia H., Dos Santos A.F., Nepachalovich P., Pedrera L., Zilka O., Inague A., Klein C., Aroua N., Kaushal K. (2024). 7-Dehydrocholesterol is an endogenous suppressor of ferroptosis. Nature.

[107] Sun W.-Y., Tyurin V.A., Mikulska-Ruminska K., Shrivastava I.H., Anthonymuthu T.S., Zhai Y.-J., Pan M.-H., Gong H.-B., Lu D.-H., Sun J. (2021). Phospholipase iPLA2β averts ferroptosis by eliminating a redox lipid death signal. Nat. Chem. Biol..

[108] Chen D., Chu B., Yang X., Liu Z., Jin Y., Kon N., Rabadan R., Jiang X., Stockwell B.R., Gu W. (2021). IPLA2β-mediated lipid detoxification controls p53-driven ferroptosis independent of GPX4. Nat. Commun..

[109] Li H., Zhu W., Zhang L., Lei H., Wu X., Guo L., Chen X., Wang Y., Tang H. (2015). The metabolic responses to hepatitis B virus infection shed new light on pathogenesis and targets for treatment. Sci. Rep..

[110] Na T.Y., Shin Y.K., Roh K.J., Kang S.A., Hong I., Oh S.J., Seong J.K., Park C.K., Choi Y.L., Lee M.O. (2009). Liver X receptor mediates hepatitis B virus X protein–induced lipogenesis in hepatitis B virus–associated hepatocellular carcinoma. Hepatology.

[111] You X., Liu F., Zhang T., Li Y., Ye L., Zhang X. (2013). Hepatitis B virus X protein upregulates oncogene Rab18 to result in the dysregulation of lipogenesis and proliferation of hepatoma cells. Carcinogenesis.

[112] Kim K.H., Shin H.J., Kim K., Choi H.M., Rhee S.H., Moon H.B., Kim H.H., Yang U.S., Yu D.Y., Cheong J. (2007). Hepatitis B virus X protein induces hepatic steatosis via transcriptional activation of SREBP1 and PPARγ. Gastroenterology.

[113] Tesfay L., Paul B.T., Konstorum A., Deng Z., Cox A.O., Lee J., Furdui C.M., Hegde P., Torti F.M., Torti S.V. (2019). Stearoyl-CoA desaturase 1 protects ovarian cancer cells from ferroptotic cell death. Cancer Res..

[114] Bartolacci C., Andreani C., Vale G., Berto S., Melegari M., Crouch A.C., Baluya D.L., Kemble G., Hodges K., Starrett J. (2022). Targeting de novo lipogenesis and the Lands cycle induces ferroptosis in KRAS-mutant lung cancer. Nat. Commun..

[115] Xu Z., Zhai L., Yi T., Gao H., Fan F., Li Y., Wang Y., Li N., Xing X., Su N. (2016). Hepatitis B virus X induces inflammation and cancer in mice liver through dysregulation of cytoskeletal remodeling and lipid metabolism. Oncotarget.

[116] You H., Wang X., Liu R., Wang Y., Zhang H., Ma L., Bao E., Zhong Y., Liu X., Kong D. (2026). HBx-mediated GPT2 suppression promotes liver cancer development by downregulating ADH1A. Cancer Biol. Med..

[117] Liu Z., Wang J., Liu L., Yuan H., Bu Y., Feng J., Liu Y., Yang G., Zhao M., Yuan Y. (2020). Chronic ethanol consumption and HBV induce abnormal lipid metabolism through HBx/SWELL1/arachidonic acid signaling and activate Tregs in HBV-Tg mice. Theranostics.

[118] Huang Q., Lei H., Ding L., Wang Y. (2019). Stimulated phospholipid synthesis is key for hepatitis B virus replications. Sci. Rep..

[119] Shi S., Zhang H., Jiang P., Zhou Y., Zhu Y., Feng T., Xie C., He H., Chen J. (2024). Inhibition of LPCAT3 exacerbates endoplasmic reticulum stress and HBV replication. Int. Immunopharmacol..

[120] Reed A., Ichu T.-A., Milosevich N., Melillo B., Schafroth M.A., Otsuka Y., Scampavia L., Spicer T.P., Cravatt B.F. (2022). LPCAT3 inhibitors remodel the polyunsaturated phospholipid content of human cells and protect from ferroptosis. ACS Chem. Biol..

[121] Zeissig S., Murata K., Sweet L., Publicover J., Hu Z., Kaser A., Bosse E., Iqbal J., Hussain M.M., Balschun K. (2012). Hepatitis B virus–induced lipid alterations contribute to natural killer T cell–dependent protective immunity. Nat. Med..

[122] Cheng X., Wang W., Zhang Z., Zhang H., Zhu P., He R., Wu M., Zhou T., Jiang Y., Jiang L. (2024). Distinctly altered lipid components in hepatocellular carcinoma relate to impaired T cell-dependent antitumor immunity. Hepatol. Int..

[123] Magarditchian G., Berest I., Ziogou A., Matsushita M., Reid M., Othman A., Kopf M. (2026). The phospholipid profile of T cells shapes ACSL4 dependency and ferroptosis sensitivity of naive, effector, and memory T cells. Proc. Natl. Acad. Sci. USA.

[124] Schmidt N.M., Wing P.A., Diniz M.O., Pallett L.J., Swadling L., Harris J.M., Burton A.R., Jeffery-Smith A., Zakeri N., Amin O.E. (2021). Targeting human Acyl-CoA: Cholesterol acyltransferase as a dual viral and T cell metabolic checkpoint. Nat. Commun..

[125] Sun S., Qi G., Chen H., He D., Ma D., Bie Y., Xu L., Feng B., Pang Q., Guo H. (2023). Ferroptosis sensitization in glioma: Exploring the regulatory mechanism of SOAT1 and its therapeutic implications. Cell Death Dis..

[126] Cassel K., Groblewski G. (2025). Long-chain mono and polyunsaturated acylcarnitines and free fatty acids cause acinar cell ferroptosis and the development of pancreatitis. Physiology.

[127] Cheng X., Tan X., Wang W., Zhang Z., Zhu R., Wu M., Li M., Chen Y., Liang Z., Zhu P. (2023). Long-chain acylcarnitines induce senescence of invariant natural killer T cells in hepatocellular carcinoma. Cancer Res..

[128] Li M., Sun R., Xu L., Yin W., Chen Y., Zheng X., Lian Z., Wei H., Tian Z. (2015). Kupffer cells support hepatitis B virus–mediated CD8+ T cell exhaustion via hepatitis B core antigen–TLR2 interactions in mice. J. Immunol..

[129] Chen Y. (2025). Ferroptosis: A Novel Target to Rescue CD8+ T Cells Against Hepatitis B Virus. Cell. Mol. Gastroenterol. Hepatol..

[130] Yao Y., Chen Z., Zhang H., Chen C., Zeng M., Yunis J., Wei Y., Wan Y., Wang N., Zhou M. (2021). Selenium–GPX4 axis protects follicular helper T cells from ferroptosis. Nat. Immunol..

[131] Matsushita M., Freigang S., Schneider C., Conrad M., Bornkamm G.W., Kopf M. (2015). T cell lipid peroxidation induces ferroptosis and prevents immunity to infection. J. Exp. Med..

[132] Wang Y., Tian Q., Hao Y., Yao W., Lu J., Chen C., Chen X., Lin Y., Huang Q., Xu L. (2022). The kinase complex mTORC2 promotes the longevity of virus-specific memory CD4+ T cells by preventing ferroptosis. Nat. Immunol..

[133] Wang W., Green M., Choi J.E., Gijón M., Kennedy P.D., Johnson J.K., Liao P., Lang X., Kryczek I., Sell A. (2019). CD8+ T cells regulate tumour ferroptosis during cancer immunotherapy. Nature.

[134] Hou C.-Y., Lv P., Yuan H.-F., Zhao L.-N., Wang Y.-F., Zhang H.-H., Yang G., Zhang X.-D. (2024). Bevacizumab induces ferroptosis and enhances CD8+ T cell immune activity in liver cancer via modulating HAT1 and increasing IL-9. Acta Pharmacol. Sin..

[135] Wang Z., Zhou L., Pang L., Qiu W., Chan A.C.-Y., Man K. (2022). IDDF2022-ABS-0176 Tumor-derived iron loaded-exosomes impair CD8+ T anti-tumor ability via ferroptosis in hepatocellular carcinoma. Gut.

[136] Liu T., Song H.-L., Zheng W.-P., Shen Z.-Y. (2015). Inhibition of HBV replication in HepG2. 2.15 cells by human peripheral blood mononuclear cell-derived dendritic cells. Ann. Clin. Lab. Sci..

[137] De Pasquale C., Campana S., Barberi C., Sidoti Migliore G., Oliveri D., Lanza M., Musolino C., Raimondo G., Ferrone S., Pollicino T. (2021). Human hepatitis b virus negatively impacts the protective immune crosstalk between natural killer and dendritic cells. Hepatology.

[138] Li N., Song X., Peng X., Li M., Zhu M., Xiao R., Wang L., Ling L., Zhao Y., Wang T. (2026). Tim-3 facilitates dendritic cell ferroptosis and impairs anti-tumor immunity in steatohepatitis-related HCC. J. Hepatol..

[139] Efimova I., Catanzaro E., Van der Meeren L., Turubanova V.D., Hammad H., Mishchenko T.A., Vedunova M.V., Fimognari C., Bachert C., Coppieters F. (2020). Vaccination with early ferroptotic cancer cells induces efficient antitumor immunity. J. Immunother. Cancer.

[140] Wiernicki B., Maschalidi S., Pinney J., Adjemian S., Vanden Berghe T., Ravichandran K.S., Vandenabeele P. (2022). Cancer cells dying from ferroptosis impede dendritic cell-mediated anti-tumor immunity. Nat. Commun..

[141] Bluml S., Kirchberger S., Bochkov V.N., Krönke G., Stuhlmeier K., Majdic O., Zlabinger G.J., Knapp W., Binder B.R., Stöckl J. (2005). Oxidized phospholipids negatively regulate dendritic cell maturation induced by TLRs and CD40. J. Immunol..

[142] Blüml S., Zupkovitz G., Kirchberger S., Seyerl M., Bochkov V.N., Stuhlmeier K., Majdic O., Zlabinger G.J., Seiser C., Stöckl J. (2009). Epigenetic regulation of dendritic cell differentiation and function by oxidized phospholipids. Blood J. Am. Soc. Hematol..

[143] Hayashi K., Nikolos F., Lee Y., Jain A., Tsouko E., Gao H., Kasabyan A., Leung H., Osipov A., Jung S. (2020). Tipping the immunostimulatory and inhibitory DAMP balance to harness immunogenic cell death. Nat. Commun..

[144] Li H., Zheng H.-W., Chen H., Xing Z.-Z., You H., Cong M., Jia J.-D. (2012). Hepatitis B virus particles preferably induce Kupffer cells to produce TGF-β1 over pro-inflammatory cytokines. Dig. Liver Dis..

[145] Zhang J., Wang Y., Fan M., Guan Y., Zhang W., Huang F., Zhang Z., Li X., Yuan B., Liu W. (2024). Reactive oxygen species regulation by NCF1 governs ferroptosis susceptibility of Kupffer cells to MASH. Cell Metab..

[146] Zong Q., Zhang H., Liu F., Li J., Liu Q., Duan Z., Duan W., Ruan M., Zhang J., Liu Y. (2024). Activation of the cGAS-STING pathway by viral dsDNA leading to M1 polarization of macrophages mediates antiviral activity against hepatitis B virus. Immunobiology.

[147] Deng Y., Lu L., Zhu D., Zhang H., Fu Y., Tan Y., Tan X., Guo M., Zhang Y., Yang H. (2024). MafG/MYH9-LCN2 axis promotes liver fibrosis through inhibiting ferroptosis of hepatic stellate cells. Cell Death Differ..

[148] Shen M., Li Y., Wang Y., Shao J., Zhang F., Yin G., Chen A., Zhang Z., Zheng S. (2021). N6-methyladenosine modification regulates ferroptosis through autophagy signaling pathway in hepatic stellate cells. Redox Biol..

